# A Fault Diagnosis Method of Four-Mass Vibration MEMS Gyroscope Based on ResNeXt-50 with Attention Mechanism and Improved EWT Algorithm

**DOI:** 10.3390/mi14071287

**Published:** 2023-06-23

**Authors:** Yikuan Gu, Yan Wang, Zhong Li, Tiantian Zhang, Yuanhao Li, Guodong Wang, Huiliang Cao

**Affiliations:** 1 School of Software, North University of China, Taiyuan 030051, China; 2 Shanxi Software Engineering Technology Research Center, Taiyuan 030051, China; 3 The General Staff, Beijing Armed Police Corps, Beijing 100027, China; 4 Beijing Institute of Aerospace Control Devices, Beijing 100039, China; 5 Key Laboratory of Instrumentation Science & Dynamic Measurement, Ministry of Education, North University of China, Taiyuan 030051, China

**Keywords:** fault diagnosis, four-mass vibration MEMS gyroscope, denoising algorithm, ResNeXt, deep neural networks, SENet

## Abstract

In this paper, a fault identification algorithm combining a signal processing algorithm and machine learning algorithm is proposed, using a four-mass vibration MEMS gyroscope (FMVMG) for signal acquisition work, constructing a gyroscope fault dataset, and performing the model training task based on this dataset. Combining the improved EWT algorithm with SEResNeXt-50 reduces the impact of white noise in the signal on the identification task and significantly improves the accuracy of fault identification. The EWT algorithm is a wavelet analysis algorithm with adaptive wavelet analysis, which can significantly reduce the impact of boundary effects, and has a good effect on decomposition of signal segments with short length, but a reconstruction method is needed to effectively separate the noise signal and effective signal, and so this paper uses multiscale permutation entropy for calculation. For the reason that the neural network has a better ability to characterize high-dimensional signals, the one-dimensional signal is reconstructed into a two-dimensional image signal and the signal features are extracted. Then, the constructed image signals are fed into the SEResNeXt-50 network, and the characterization ability of the model is further improved in the network with the addition of the Squeeze-and-Excitation module. Finally, the proposed model is applied to the FMVMG fault dataset and compared with other models. In terms of recognition accuracy, the proposed method improves about 30.25% over the BP neural network and about 1.85% over ResNeXt-50, proving the effectiveness of the proposed method.

## 1. Introduction

In recent years, microelectromechanical systems (MEMS) sensors [1] have played a key role in many application areas, such as inertial navigation systems [2,3], automotive safety systems [4], aerospace systems [5], and smart electronics. Although MEMS sensors are designed and manufactured under strict quality control and testing [6,7], failures are still unavoidable due to uncertainties in the operating environment, device aging, and manufacturing defects, and incorrect sensor measurements can lead to serious consequences and even endanger personal safety. First, the complex and diverse operating principles and internal structures of MEMS sensors [8] make fault diagnosis complex and difficult. Second, sensor failures [9] are usually manifested as small performance changes, which are difficult to detect by traditional methods or direct observation. Therefore, certain techniques and methods are needed to improve the accuracy and reliability of fault diagnosis.

Traditional signal processing methods, as one of the most traditional fault diagnosis methods, usually use filtering [10]. Shang et al. used an adaptive signal feature processing approach to extract effective features of the signal based on the MCEEMDAN method [11]. Shi et al. used wavelet threshold denoising and wavelet packet threshold denoising methods for high-G MEMS accelerometer sensors using calibration analysis to improve the performance and accuracy of the sensor [12]. Jiang et al. proposed a method to extract seismic wavefield information based on the adaptive local singular value decomposition (ALSVD) method. The authors divided the data into different local regions, decomposed and reconstructed the data in each local region, and extracted the effective information of the seismic wavefield [13]. Sun et al. proposed an improved multichannel singular spectrum analysis and frequency–spatial domain decomposition for comprehensive fault diagnosis of rolling bearings, which improved the accuracy and reliability of fault diagnosis [14]. The signal processing-based method can capture the direct features of the signal. However, signal processing methods are sensitive to noisy environments and have difficultly processing complex nonlinear signals, so they have limited accuracy and robustness in complex fault diagnosis scenarios.

In recent years, fault diagnosis methods that use machine learning algorithms [15] to train and classify sensor data have shown significant advantages [16]. As a supervised learning algorithm, SVM [17] is able to handle complex fault patterns and nonlinear relationships with strong generalization and adaptiveness, but it is computationally complex and not suitable for classification for a large number of classes [18]. Wang et al. proposed a fault detection and diagnosis method based on adaptive models and a two-layered random forest, allowing the system to better cope with variations and uncertainties in real-world operating conditions [19]. Fang et al. proposed an adaptive multiscale and dual subnet convolutional auto-encoder (AMDSCAE) to detect intermittent faults in analog circuits in noisy environments. Although the proposed AMDSCAE achieves high accuracy in the detection of intermittent faults in analog circuits, it is slightly more expensive than other networks in terms of computational cost [20]. Gao et al. proposed an automatic fault detection method for seismic images based on a novel multiscale attention convolutional neural network (MACNN), which can effectively extract fault features and automate fault detection by introducing a multiscale attentional mechanism [21].

In this paper, the basic working principle of a four-mass vibration MEMS gyroscope (FMVMG) and the common types of faults, and the traditional methods of signal processing combined with machine learning algorithms to propose the IEWT-SEResNeXt algorithm are introduced. Among them, IEWT essentially combines the EWT algorithm with multiscale permutation entropy, decomposes the original signal into mra components, rejects the noisy components by calculating the multiscale permutation entropy value of each component, and finally obtains the reconstructed signal. This stage performs preliminary filtering of the signal to remove the high-frequency white noise in the original signal and prepares it for subsequent input into the neural network. SEResNeXt is an improved network that introduces the SE (Squeeze-and-Excitation) attention mechanism module into the ResNeXt network. The addition of the SE module to the ResNeXt network enables the network to adaptively adjust the importance of features between channels and strengthen the representational and generalization capabilities of the network. Comparing the method proposed in this paper with the BP neural network and SEResNeXt, it is shown by experimental results that the method mentioned in this paper has a high recognition rate of the fault signal of FMVMG. Compared with other models, it does have different degrees of improvement and proves its effectiveness.

The structure of this paper is as follows: Section 2 will introduce the structure and common fault types of FMVMG; Section 3 describes the method proposed in this paper; Section 4 proves its effectiveness through experiments; Section 5 summarizes the work of this paper.

## 2. Structure of the Four-Mass Vibration MEMS Gyroscope and Common Types of Faults

### 2.1. Structure of the Four-Mass Vibration MEMS Gyroscope

**Figure 1:**
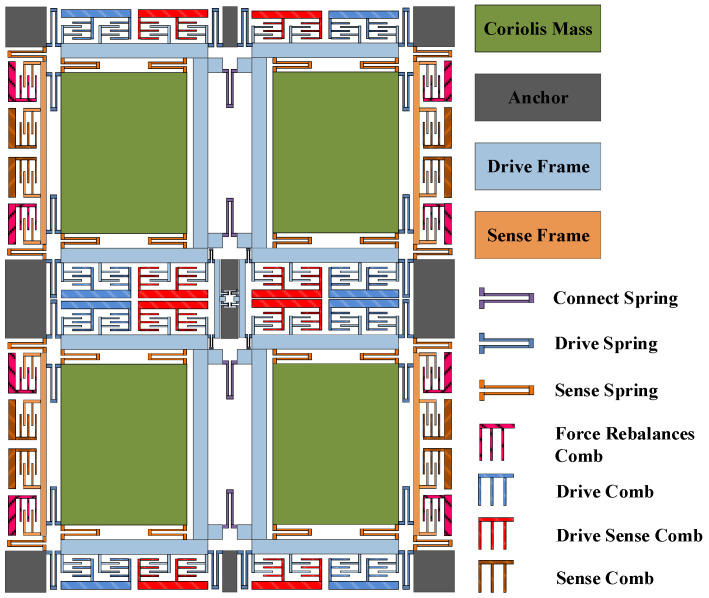
The overall structure of FMVMG.

The overall structure of the four-mass vibration silicon micro gyroscope is shown in Figure 1, which is drawn by Solidworks2016, ANSYS19.0, matlab2023a and PyCharm2021.1.1. The structure adopts the design principle of symmetric design and structural decoupling, which has four symmetric structures, support beams, and anchor points. The symmetric structure consists of Coriolis masses, driving comb frames, driving electrodes and combs, driving support beams, driving detection combs, detection comb frames, detection electrodes and combs, detection support beams, and force balance combs. The gyroscopic structure is connected by the anchor points of support beams so that it is suspended above the glass substrate, and its driving and detecting combs are working by means of sliding film combs.

**Figure 2:**
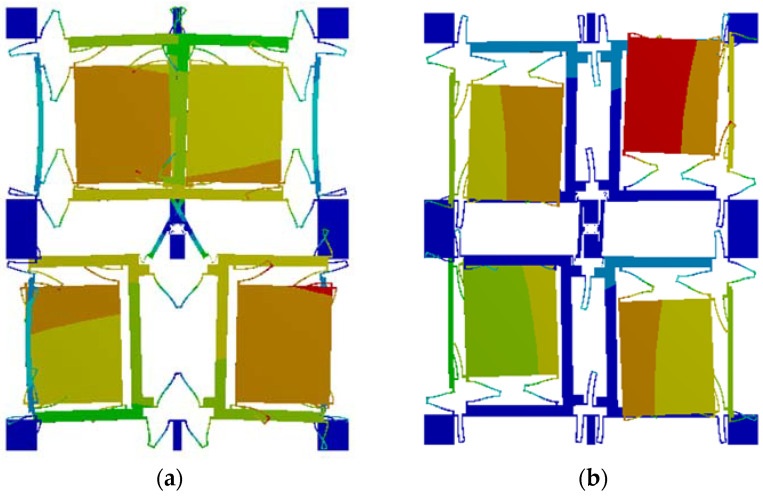
(**a**) Driving mode; (**b**) sensing mode.

Figure 2 shows the structural simulation by ANSYS19.0. As shown in Figure 2a, when a DC bias with AC voltage is given to driving electrodes, the four-mass block moves due to the electrostatic force, and its driving mode is the same frequency reverse motion on the X-axis. When there is an input angular velocity on the Z-axis, the detection mode is generated by the Coriolis effect. The motion state is shown in Figure 2b as the same frequency inverse motion on the Y-axis.

### 2.2. Common Types of Faults

**Figure 3:**
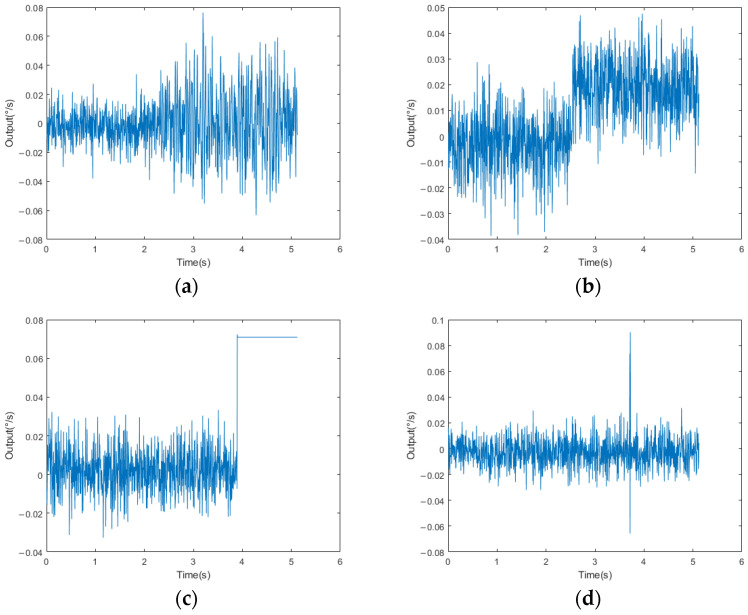
(**a**) Scale Factor Fault; (**b**) Bias Fault; (**c**) Device Fault; (**d**) Transient Fault.

The FMVMG is subject to failure, just like any other technology, and is usually affected by various factors, such as manufacturing defects, mechanical stress, environmental effects, or improper use. In the manufacturing process of FMVMG, the main types of failure data collected are divided into the following four types, which are drawn by matlab2023a: (Figure 3):

(1) Scale Factor Fault:

The scale factor of the gyroscope refers to the proportional relationship between the input angular velocity and the output signal. This fault can lead to the amplification or reduction in the output signal, which results in the error of angular velocity measurement. This may be caused by damage to the internal components of the sensor or by errors in the manufacturing process. If the proportionality factor is wrong, it may cause the amplitude of the output signal to become larger, resulting in a proportional mismatch between the output signal and the actual angular velocity.

(2) Bias Fault:

When the zero-offset value of the gyroscope changes, it will cause the gyroscope to have a fixed offset in the nonrotating state. This fault may be caused by poor internal circuitry of the sensor or interference from the external environment. If this happens, check the power supply of the gyroscope and calibrate the gyroscope.

(3) Device Fault:

Device failure may be caused by component damage, circuit failure, or connection problems. This failure may lead to complete signal loss or abnormal output signal, and the output signal cannot correctly reflect the angular velocity change.

(4) Transient Fault:

The gyroscope may experience a transient fault, such as power supply noise, electromagnetic interference, or overvoltage. This transient fault may cause the output signal to suddenly become larger at a certain moment. Unstable supply voltage or a problem with the power supply line may also result in a particularly large output value for a certain number.

## 3. Model and Algorithm

### 3.1. Empirical Wavelet Transform

On the basis of EMD, Gilles proposed a new theory of adaptive wavelet analysis in 2013. This method empirically segments the Fourier frequency domain of a signal with noise to determine the frequency range of the band-pass filter, adaptively processes the filter bank, extracts different components through the filter bank, and represents the components as time-domain modal signals corresponding to each frequency band. The theoretical analysis process of EWT is shown below.

Based on the spectrogram of a given signal *f*(*t*), the Fourier spectrum is adaptively defined as [0,*π*], and assumed to be divided into *N* consecutive sections, denoted as Λ*_n_* = [ω*_n_*−_1_,ω*_n_*], *n* = 1,2,…,*N*, where ω_0_ = 0; ω*_n_* = *π*, and the empirical wavelet is defined as a band-pass filter on each section. The interval of the entire Fourier spectrum is shown in Equation (1):

(1) $$\sum_{n=1}^{N}\Lambda_{n}=[0,\pi ]$$

Gilles designed the empirical wavelet theory using the idea of constructing Littlewood–Paley and Meyer wavelets.

First, the empirical wavelet function *ψ_n_*(*ω*) and the empirical scale function *φ_n_*(*ω*) are represented as shown in Equations (2) and (3), respectively:

(2) $$\psi_{n}(\omega )=\left\{\begin{array}{ll} 1, & \omega_{n}+\tau_{n}\leq \left|\omega \right|\leq \omega_{n+1}-\tau_{n+1}\\ \cos\left[\frac{\pi }{2}\beta \left(\frac{1}{2\tau_{n+1}}(\left|\omega \right|-\omega_{n+1}+\tau_{n+1})\right)\right], & \omega_{n+1}-\tau_{n+1}\leq \left|\omega \right|\leq \omega_{n+1}+\tau_{n+1}\\ \sin\left[\frac{\pi }{2}\beta \left(\frac{1}{2\tau_{n}}(\left|\omega \right|-\omega_{n}+\tau_{n})\right)\right], & \omega_{n}-\tau_{n}\leq \left|\omega \right|\leq \omega_{n}+\tau_{n}\\ 0, & others \end{array}\right.$$

(3) $$\varphi_{n}(\omega )=\left\{\begin{array}{ll} 1, & \left|\omega \right|\leq \omega_{n}-\tau_{n}\\ \cos\left[\frac{\pi }{2}\beta \left(\frac{1}{2\tau_{n}}(\left|\omega \right|-\omega_{n}+\tau_{n})\right)\right], & \omega_{n}-\tau_{n}\leq \left|\omega \right|\leq \omega_{n}+\tau_{n}\\ 0, & others \end{array}\right.$$

where *β*(*x*) = *x*^4^(35 – 84*x* + 70*x*^2^ + 20*x*^3^).

Next, the empirical wavelet coefficients are obtained by the inner product of detail coefficients $W_{f}^{e}$(*n*, *t*) and approximation coefficients $W_{f}^{e}$(0, *t*), as shown in Equations (4) and (5):

(4) $$W_{f}^{e}(n,t)=\langle f,\psi_{n}\rangle =\int f(\tau )\bar{\psi_{n}(\tau -t)}d\tau =F^{-1}\left[f(\omega )\bar{\psi_{n}(\omega )}\right]$$

(5) $$W_{f}^{e}(0,t)=\langle f,\varphi_{1}\rangle =\int f(\tau )\bar{\varphi_{1}(\tau -t)}d\tau =F^{-1}\left[f(\omega )\bar{\varphi_{1}(\omega )}\right]$$

where *ψ_n_*(*ω*) and *φ_n_*(*ω*) are the Fourier transforms of *ψ_n_*(*t*) and *φ_n_*(*t*), *F*[·] and *F*^−1^[·] are the Fourier transform and the inverse transform, respectively.

Finally, the reconstructed signal can be expressed as shown in Equation (6):

(6) $$\begin{array}{ll} f(t) & =W_{f}^{e}(0,t)*\varphi_{1}(t)+\sum_{n=1}^{N}W_{f}^{e}(n,t)*\psi_{n}(t)\\ & =\left(W_{f}^{e}(0,\omega )\varphi_{1}(\omega )+\sum_{n=1}^{N}W_{f}^{e}(n,\omega )\psi_{n}(\omega )\right) \end{array}$$

This leads to the decomposition of the original signal to obtain the modal component *f_k_*(*t*), (*k* = 1, 2, …) from high to low frequency:

(7) $$\left\{\begin{array}{l} f_{0}(t)=W_{f}^{e}(0,t)*\varphi_{1}(t)\\ f_{k}(t)=W_{f}^{e}(k,t)*\psi_{k}(t) \end{array}\right.$$

### 3.2. Multiscale Permutation Entropy

Alignment entropy is a metric used to measure the complexity of time series and introduces the idea of alignment when calculating the complexity between reconstructed subsequences. This method has the advantages of simple and fast calculation, strong noise immunity, and suitability for online monitoring. In this paper, PE was used to calculate each modal component and divide the noise component and the effective component by PEs. The process of PE description is as follows.

Given a time series {*x*(*i*), *i* = 1, 2, …, *N*}, and reconstructing the series, the reconstruction is shown in Equation (8):

(8) $$\left[\begin{array}{llll} x(1) & x(1+\tau ) & \cdots & x(1+(m-1)\tau )\\ \vdots & \vdots & \cdots & \vdots \\ x(j) & x(j+\tau ) & \cdots & x(j+(m-1)\tau )\\ \vdots & \vdots & \cdots & \vdots \\ x(N-(m-1)\tau ) & x(N-(m-2)\tau ) & \cdots & x(N) \end{array}\right]$$

where *m* is the cut-in dimension and *τ* is the delay time. In the reconstruction matrix, each row of the sequence can be considered as a reconstruction vector, and there are *N* − (*m* − 1)*τ* reconstruction vectors in total. By arranging the values in each sequence in ascending order, Equation (9) is obtained:

(9) $$x(j+(j_{1}-1)\tau )\leq x(j+(j_{2}-1)\tau )\leq \cdots \leq x(j+(j_{m}-1)\tau )$$

where *j*_1_, *j*_2_, …, *j_m_* denotes the *m*th index of the *j*th reconstructed vector.

For the reconstructed vectors *x*(*j*) in the reconstructed matrix, the alignments are aligned to obtain a sequence *S*(*l*) = [*j*_1_, *j*_2_, …, *j_m_*], where *l* = 1, 2, …, *k*, *k* ≤ *m*!.

Among most *m*! different sequences, the probability *P_k_* of occurrence of each sequence is calculated. The permutation entropy is defined as the Shannon entropy of *k* sequences as shown in Equation (10):

(10) $$PE(m)=-\sum_{l=1}^{k}P_{l}\ln P_{l}$$

The sample entropy of a single-scale sequence is not enough to express the information of the signal, and so the original signal *x*(*i*) is coarsely granularized. The coarse granulation process is as in Equation (11):

(11) $$\begin{array}{cc} y_{l}(i)=\frac{1}{l}\sum_{j=(i-1)l+1}^{il}x(j) & i=1,2,\cdots ,\frac{N}{l} \end{array}$$

where *l* is the coarse granulation factor and *y_l_*(*i*) is the coarse granulation signal under coarse granulation factor *l*.

### 3.3. Image Reconstruction Method

The signal reconstructed by EWT is still a one-dimensional time series. Analyzing the signal from a one-dimensional perspective alone is not enough to highlight the characteristics of the signal, and at the same time, in order to input the signal samples into the neural network, the one-dimensional time series needs to be reconstructed into a two-dimensional image to improve the accuracy of the model judgment.

In this paper, the signal segment *x*(*i*), *i* = 1, 2, …, *m*^2^ is taken and the sequence *x*(*i*) is normalized according to Equation (12), where max(·) denotes the maximum value in the sequence and min(·) denotes the minimum value in the sequence.

(12) $$x(i)=\frac{x(i)-\min(x)}{\max(x)-\min(x)}$$

**Figure 4:**
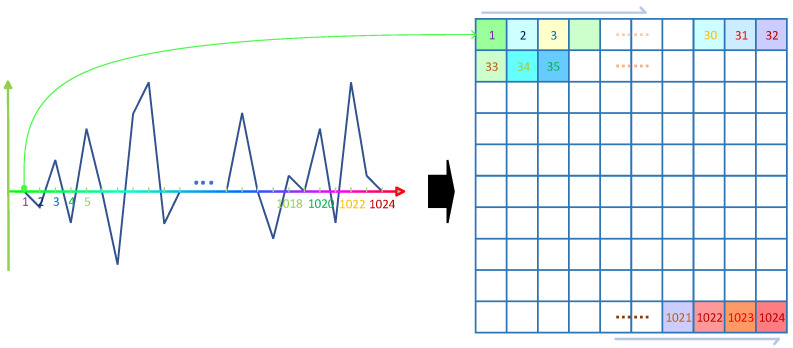
Reconstruction process.

**Figure 5:**
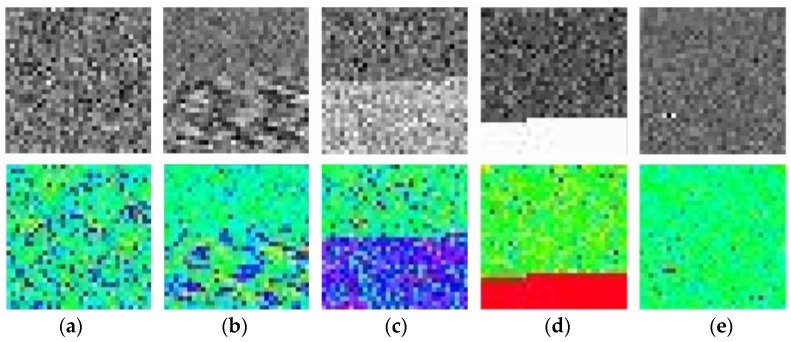
Image sample. (**a**) Normal; (**b**) Scale Factor Fault; (**c**) Bias Fault; (**d**) Device Fault; (**e**) Transient Fault.

The normalized time series is reconstructed into an m × m two-dimensional matrix and a gray image is generated. The reconstruction process is shown in Figure 4, and to increase the image informativeness, the generated grayscale image is converted to a pseudo-color image as shown in Figure 5. In this paper, m was taken as 32.

### 3.4. SEResNeXt-50

**Figure 6:**
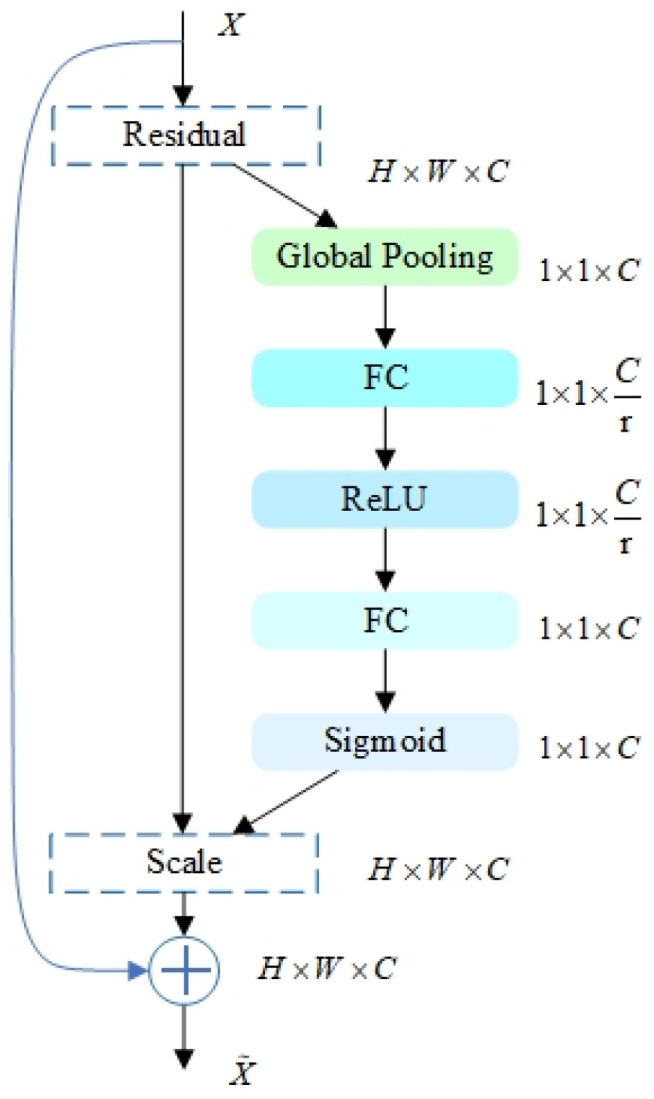
The SEResNeXt module.

**Figure 7:**
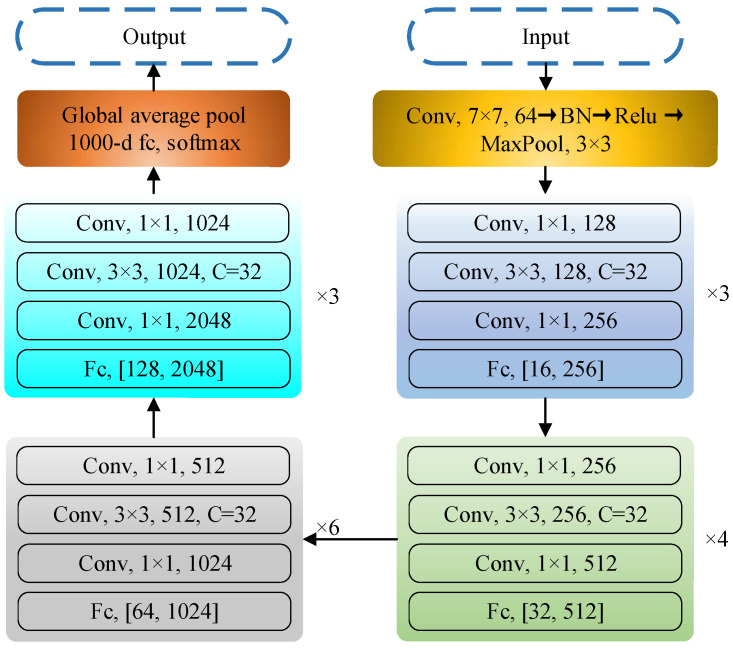
Overall framework diagram.

SENet, the champion model of ImageNet 2017, was proposed by Jie Hu et al. [22] through the “Squeeze-and-Excitation (SE)” module, which dynamically adjusts the response value between channels of the model and assigns the importance between channels. ResNeXt [23] combines block and split-transform-merge strategies by learning the advantages of VGG and ResNet. SEResNeXt retains the multibranch structure of ResNeXt and improves the performance of deep learning by introducing the attention mechanism of the SE module and keeps the number of parameters low. As shown in Figure 6, the SE module is applied to the residual branch. The overall framework diagram of the network is shown in Figure 7.

**Figure 8:**
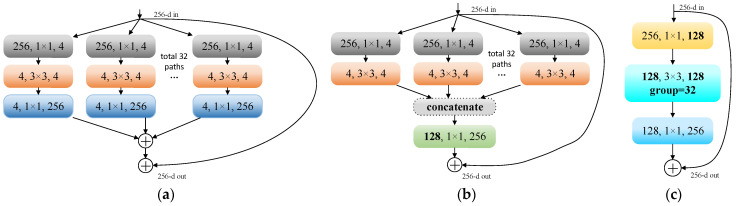
(**a**) The original form of the ResNeXt module; (**b**) equivalent type of GoogleNet; (**c**) equivalent type of group convolution.

The biggest improvement of SEResNeXt over SEResNet is the introduction of the cardinality structure, which facilitates feature interaction between branches by introducing covariant connections between branches, as shown in Figure 8.

In order to balance the feature expression capability between each branch, the allocation of the number of convolutional kernels is adjusted.

Moreover, in order to maintain a comparable number of parameters to that of conventional ResNet, the *C* and *d* parameters in the ResNeXt block are determined by Equation (13) at the corresponding number of parameters:

(13) C⋅(256⋅d+3⋅3⋅d⋅d+d⋅256)

In the SE module, the Squeeze operation is performed firstly, by a global average pooling operation, and compresses the spatial dimension of each input feature map from H × W to 1. This operation compresses the features by spatial dimension, converting each 2D feature channel to a real number. This real number is the one that has a global perceptual field, and the output dimension matches the number of input feature channels. The computation is performed as in Equation (14):

(14) $$z_{c}=F_{sq}(u_{c})=\frac{1}{H\times W}\sum_{i=1}^{H}\sum_{j=1}^{W}u_{c}(i,j)$$

Next, Excitation and Reweight operations are performed to calculate their channel weights using channel relations and are multiplied onto each channel to complete the recalibration of each feature channel.

### 3.5. Step of iEWT-SEResNeXt-50

**Figure 9:**
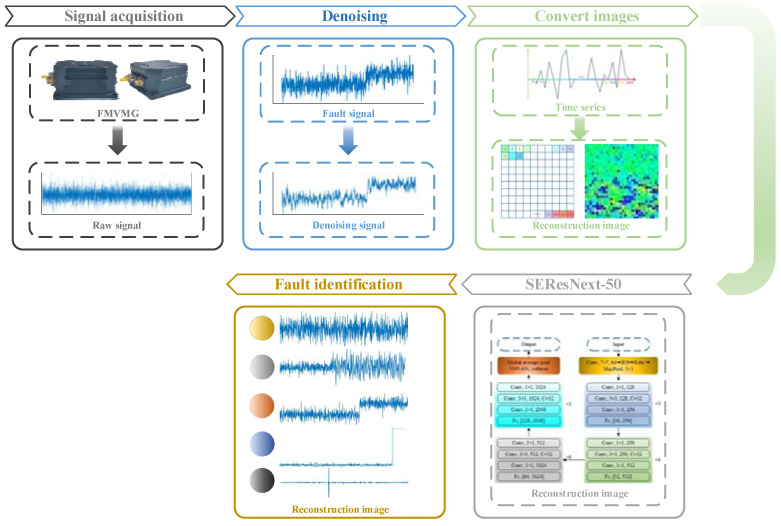
Steps of iEWT-SEResNeXt-50.

In order to further improve the accuracy of the machine learning model, this paper combined the signal processing method iEWT with the machine learning algorithm SEResNeXt and applied it to the FMVMG. The specific processing steps (as in Figure 9) are as follows:

(1) Signal acquisition

The output signals of the FMVMG are collected under different working conditions, as well as the signal data under which different faults occur, and the fault signals are cropped to the same interval length, which is set to 1024 in this paper.

(2) Denoising

The iEWT decomposition is performed on the faulty signal of FMVMG, and the multiscale permutation entropy of the mra component is calculated and the scale factor is set to 4, m = 3, r = 1. The denoised signal is obtained by reconstructing the decomposed signal.

(3) Convert images

The denoised signal is subjected to the reconstruction operation to construct a two-dimensional image signal. Compared with one-dimensional signals, neural networks show greater advantages for the processing of two-dimensional signals. Therefore, it is necessary to up-dimension the one-dimensional signal. In this paper, a 32 × 32 matrix was constructed, and the values within the matrix were normalized to construct grayscale images, which was next converted to a pseudo-color image.

(4) SEResNeXt-50

The train set is fed into the SEResNeXt-50 network for model training. The SGD optimizer was used by default to update the parameters in this paper.

(5) Fault identification

The test set is fed into the SEResNeXt-50 model for testing and the test results are tallied.

## 4. Experiment

**Figure 10:**
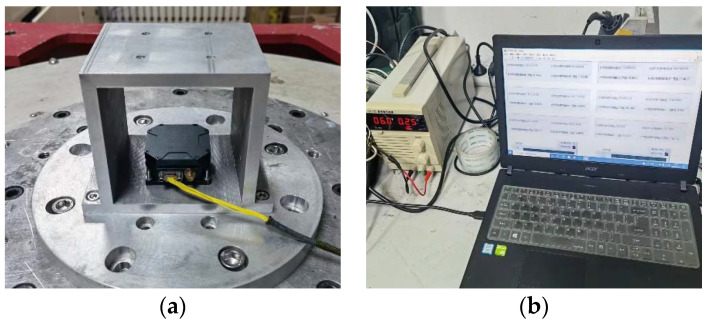
(**a**) Platform; (**b**) laptop and power supply.

In this part, a FMVMG prototype was used for the acquisition of experimental data, which was fixed on a stationary horizontal platform to acquire signals. The device acquired the motion state of the gyroscope with a sampling frequency of 200 Hz, and the experimental environment is shown in Figure 10. The dataset of this experiment contains the signal output of this device under the normal condition and four fault conditions, which are Normal, Scale Factor Fault, Bias Fault, Device Fault, and Transient Fault.

In order to improve the performance and accuracy of the model and cover the variability of the samples, this experiment expands the dataset by slider sampling of the collected fault data with a slider segment length of 1024, while achieving more comprehensive data coverage. The distribution of the number of the final dataset is shown in Table 1.

**Figure 11:**
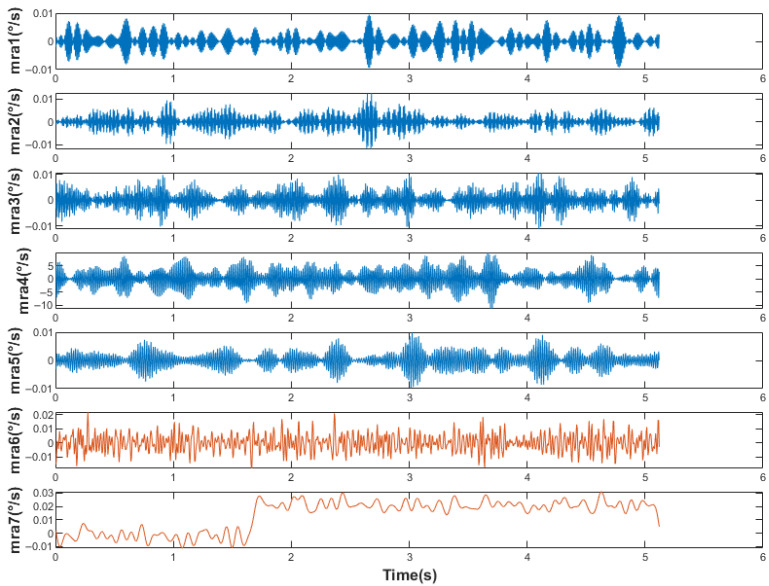
Decomposition process.

**Figure 12:**
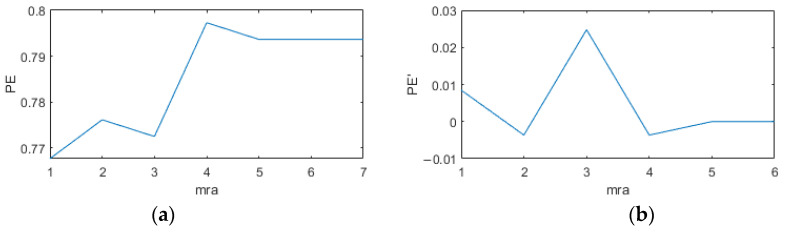
The derivation process for the components. (**a**) Multiscale alignment entropy; (**b**) derivation process.

**Figure 13:**
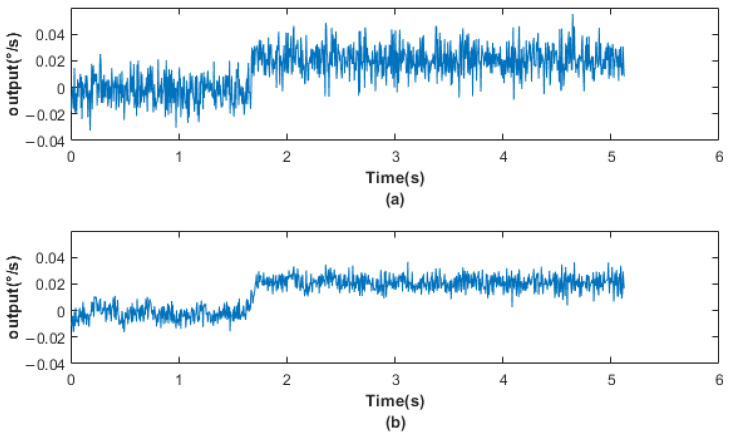
(**a**) Original signal; (**b**) reconstructed signal.

First, the original signal was denoised according to the IEWT algorithm, which decomposes the signal in the process as shown in Figure 11. For its decomposition result mra, if the number of mra is 2, the first component is considered as the noisy signal and the second component as the valid signal. If the number of mra is greater than 2, its respective multiscale permutation entropy is calculated, and PEs are obtained. Derive PEs, and the position of the index number +1 of the last extreme point of the PEs is considered as the dividing point, and the component after the dividing point is the valid signal. The multiscale permutation entropy of the signals in Figure 10 is calculated as shown in Figure 11, and according to Figure 12b, the 5th position is considered as the demarcation point. The 6th and 7th components are considered as valid signals. The reconstructed signal was compared with the original signal, as shown in Figure 13. The reconstructed signal was applied to the method in Section 3.3, and the reconstructed image is shown in Figure 5.

The total number of images in the dataset was 10,000, and the number of training set and test set were divided into 8000 and 2000 according to the ratio of 4:1, and each classification included 1600 training set images and 400 test set images, respectively, and the order of all images was disordered. Training was performed using PyCharm2021.1.1, in which the initial learning rate was set to 0.001.

**Figure 14:**
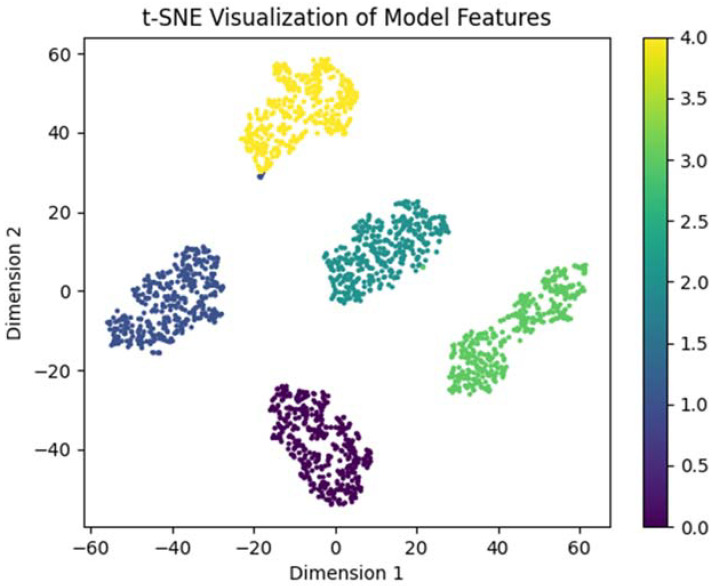
Feature visualization.

After 100 epochs of training, the model has converged, and the average accuracy is 99.40% after combining the results of 5 training sessions. Figure 14 shows the diagnostic results of the final model of iEWT-SEResNeXt-50 in this paper using the t-sne model visualization technique. The figure shows that the model has clear separation between categories and tighter clusters, and can be accurately identified for the vast majority of samples.

**Figure 15:**
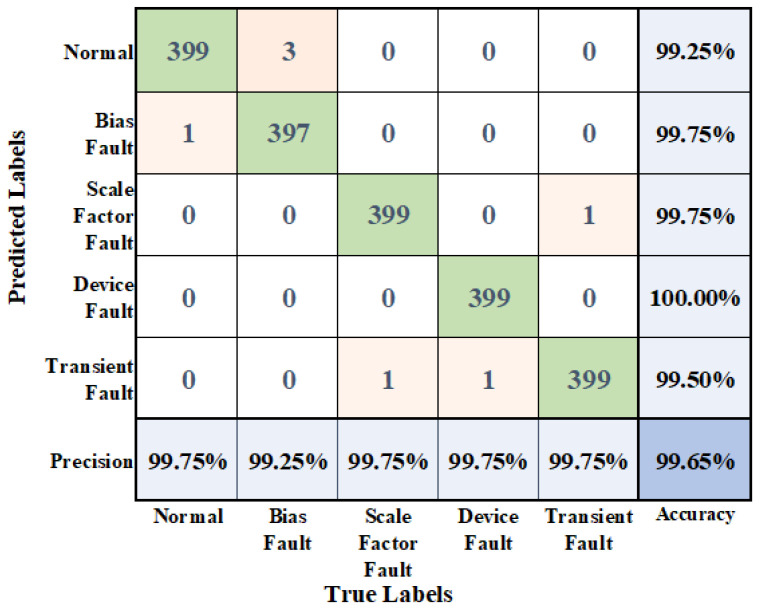
Confusion matrix.

To show the experimental results in more detail, a confusion matrix was drawn to visualize the classification results. The visualization results are shown in Figure 15, where it can be seen that the average recall rate is 99.65% and the precision rate is 99.65%.

To further demonstrate the diagnostic capability of the iEWT-SEResNeXt-50 model, the proposed method was compared with other methods (BP neural network and ResNeXt-50) by averaging the data from five experiments under the same conditions, and the comparison results are shown in Table 2. Compared with the BP neural network, the proposed method in this paper has a large improvement. Meanwhile, there is a certain gap between ResNeXt-50 and iEWT-SEResNeXt-50, which may be due to the complexity of discrete signals, and the diagnosis results do improve to some extent after the denoising algorithm and attention mechanism module are added. Therefore, it can be proved that the method in this paper has some rationality.

## 5. Conclusions

This paper firstly introduced the basic principles of the four-mass vibrating gyro and several common classical fault types, followed by a fault diagnosis algorithm using a signal processing algorithm combined with machine learning. The improved EWT algorithm was combined with SEResNeXt-50 to perform the fault identification task on the output signal of the introduced FMVMG. Since the output signal had a large amount of white noise and the complex discrete signal tended to interfere with the identification results, this paper used the iEWT algorithm to decompose the original signal. To facilitate processing by deep learning models, the obtained reconstructed signal was up-dimensioned to obtain the color image signal. The image dataset was trained using the SEResNeXt-50 network, and the obtained model was used for the fault diagnosis work of FMVMG. After several experiments, it was concluded that the average accuracy of the model on the self-built dataset was 99.40%. The proposed method in this paper was then compared with other methods, and the results showed that the recognition accuracy of the method in this paper was improved by 30.25% compared with the BP neural network and 1.85% compared with ResNeXt-50, which proved that the introduction of the signal processing algorithm and attention mechanism module did have better fault recognition capability.

## Figures and Tables

**Table 1:** The amount of data.

Failure Type	Size
Normal	2000
Scale Factor Fault	2000
Bias Fault	2000
Device Fault	2000
Transient Fault	2000

**Table 2:** The comparison results.

Method	Average Accuracy	Precision	Recall	F1 Core	References
BP neural networks	73.80%	73.62%	73.80%	73.18%	[24]
ResNeXt-50	97.18%	97.20%	97.18%	97.18%	[23]
iEWT-SEResNeXt-50	99.40%	99.40%	99.40%	99.39%	-
